# Case Report: Severe Aortic Valve Regurgitation and Pseudoaneurysm in Aortic Valve-Sparing Operation: The Usefulness of Multimodality Imaging in a Complex Clinical Scenario

**DOI:** 10.3389/fcvm.2021.719405

**Published:** 2021-08-25

**Authors:** Leonardo Varotto, Luca Spigolon, Alberto Dotto, Valentina Siviero, Marta Scodro, Ester Cabianca, Paolo Magagna, Francesco Caprioglio

**Affiliations:** ^1^Department of Cardiology, San Bortolo Hospital, Vicenza, Italy; ^2^Department of Radiology, San Bortolo Hospital, Vicenza, Italy; ^3^Department of Cardiac Surgery, San Bortolo Hospital, Vicenza, Italy

**Keywords:** aortic regurgitation, Tirone-David operation, transcatheter aortic valve implantation, aortic pseudo-aneurysm, three-dimensional printed model

## Abstract

**Background:** Failure of the native aortic valve and degenerative anatomy of ascending aorta in patients with previous Tirone-David operation may represent a clinical challenge, because sometimes the risk of reoperation is prohibitive.

**Case:** We described the case of a patient suffering from severe aortic valve regurgitation and pseudoaneurysm of the aortic arch, 6 years after cardiac surgery operation. The aim of this clinical case was to assess if the complex anatomy of aortic pseudoaneurysm and aortic root geometry can be accurately reproduced from contrast-enhanced computed tomography scan into a three-dimensional (3D) printed model. Based on this procedural method, with the aid of transesophageal 3D ultrasound, we efficaciously treated the patient percutaneously with a combination of transcatheter occluder device plus microcoil embolization and transfemoral aortic valve implantation. The patient was free from complications and the need to redo cardiac surgery.

**Conclusion:** To the best of our knowledge, this is the first description of two simultaneous complications and their staged treatment in a patient with previous aortic valve-sparing operation. This is a useful report in a single 3D model applying such specific technology to these two simultaneous clinical settings.

## Introduction

The development of aortic valve-sparing operations (reimplantation of the aortic valve and remodeling of the aortic root) expanded the surgical armamentarium for treating patients with aortic root dilation caused by a variety of disorders (1).

The main problems with aortic valve sparing (AVS) are the development of aortic insufficiency (AI) after surgery and, along the suture with the Dacron graft, the size and shape of the remaining degenerative ascending aorta. These problems can cause the need for reoperation of the aortic valve. Because AI after AVS is “native valve AI,” many clinicians do not refer the patient back to the surgeon for reoperation until symptoms or signs of ventricular dysfunction develop.

Redoing aortic valve surgery carries a higher mortality and morbidity compared with first time transcatheter aortic valve implantation (TAVI) and often requires, if associated with other problems, concomitant complex procedures (2, 3).

To our knowledge, there are few reports of TAVI in a patient with severe AI after a valve-sparing ascending aortic repair (4, 5), but none with a concomitant aortic arch pseudoaneurysm (AAP).

## Case Description

**Figure 1:**
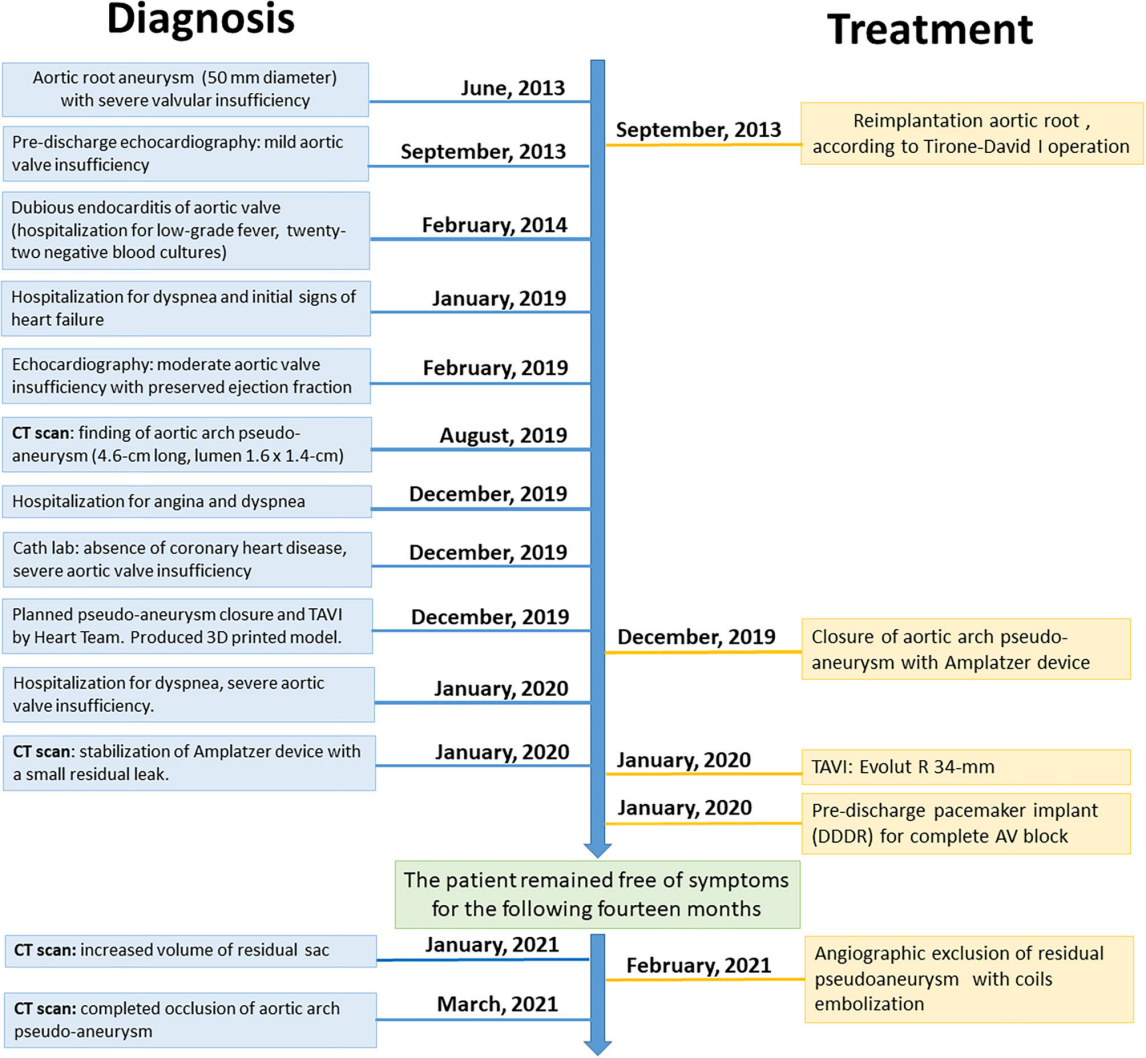
Timeline of the reported clinical case.

A 68-year-old man with degenerative aortic dilatation underwent an ascending aorta aneurysm repair with a Tirone-David I valve-sparing procedure, using a 30-mm Gelweave graft (Vascutek, Inchinnan Renfrewshire, Scotland, United Kingdom) 6 years previously (Figure 1).

Unfortunately, in addition to chronic obstructive pulmonary disease (COPD) with obstructive sleep apnea syndrome (OSAS) and cerebral ischemic outcomes, he developed progressively severe AI and repeated episodes of heart failure. The heart team recommended TAVI over open surgery because of the risk for respiratory failure and chronic cerebral vasculopathy.

**Figure 2:**
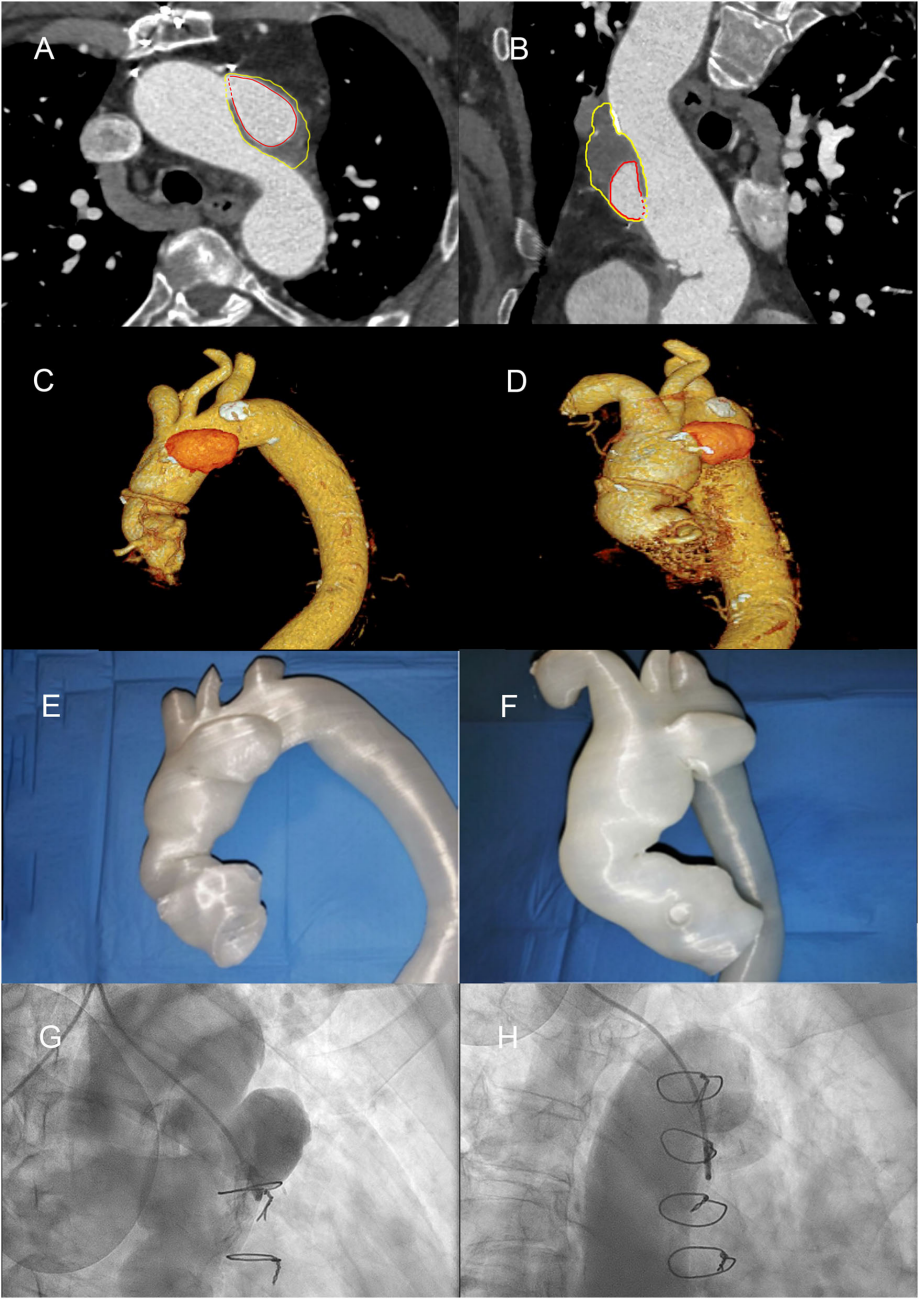
Diagnostic angiography, computed tomography scans, and 3D printed thoracic aorta model, demonstrating aortic root, and aortic arch pseudoaneurysm (AAP). **(A)** Computed tomography (CT) angio-study, axial image, showing pre-treatment of AAP. The red line highlights the perfused part of the AAP; the yellow line highlights the thrombosed part. **(B)** CT angio-study, curved multiplanar reconstruction (MPR) of AAP. **(C,D)** Volume rendering (VR) reconstructions of ascending aorta. **(E,F)** 3D print of the thoracic aorta and AAP. **(G)** Right anterior oblique (RAO) cranial angiographic image of AAP. **(H)** RAO caudal angiographic image of AAP.

Computed tomography (CT) confirmed that transfemoral access was possible, but revealed a pseudoaneurysmatic sacciform dilation at the small curve of the aortic arch (Figures 2A–D) probably originating from the arterial cannulation site used for cardiopulmonary bypass (extracorporeal circulation site—ECC site) in the remaining degenerated aortic wall.

## Diagnostic Assessment

On CT, the perimeter of the native annulus measured 91 mm, and the Gelweave graft diameter was 30 mm, with a length of 7.5 cm.

The main challenges were the choice of the TAVI bioprosthesis (supra-annular vs. intra-annular, sizing of the prosthesis, lack of calcification, and possible mobility of the device due to aortic valve insufficiency) and how to close the AAP (using percutaneous device, covered stent, or thrombin injection/glue?). Finally, we considered with the help of the 3D printed model above all the possible interferences between the bioprosthesis, the percutaneous occluder, the aortic prosthesis in Dacron, and the emergence of the epiaortic vessels. Contrast-enhanced cardiac CT scans from the patient were post-processed and produced as 3D printed thoracic aorta model of the AAP and aortic root (Figures 2E,F). The transverse diameter was measured at six anatomical landmarks, compared across three stages: the original contrast-enhanced CT images with volumetric reconstruction, the stereolithography format computerized model prepared for 3D printing, and the contrast-enhanced CT of the 3D printed model (immersed in a plastic container filled with ~80 ml Ultravist and 2.2 L of water).

Due to complex anatomy in the aortic region, in particular, the area of aortic root and aortic arch with a number of important arterial branches arising from it, it was difficult to appreciate the real 3D relationship between aortic disease and these arterial branches with conventional CT images.

Since only deidentified CT images were used for the generation of the 3D printed model, definitive 3D printed model ethical approval and patient informed consent were waived due to the retrospective nature of data collection.

No report is available in the literature with regard to the use of 3D printing, in producing physical models of the aorta accurately, in a patient with AVS and pseudoaneurysm involving the aortic arch. The rationale for carefully studying these two pathologies in this clinical case is that both aortic pathologies represent common cardiovascular disease, which is associated with high morbidity and mortality.

A 3D reconstruction of the aorta revealed a 6 × 2.8 × 3.9-cm pseudoaneurysm (volume 27 ml) arising from the aortic arch (perfused diameter 4.1 × 2.2 × 2.6 cm, volume 13 ml), with a diameter orifice of 1.6 × 1.4 cm. The dimension of the aortic root revealed a 30-mm diameter, with annulus of 26 × 31 mm diameter (89.6 mm perimeter) and left ventricular outflow tract area of 5.5 cm^2^.

The 3D printing was a valid support in demonstrating the 3D relationship between the aortic root, AAP, and arterial branches and provided a clear guidance for clinical management of these life-threatening cardiovascular diseases, by providing a preoperative training and simulation of percutaneous/endovascular approach with a customized occluder device.

The 3D prototype was first examined in a hybrid room under fluoroscopy, allowing us to obtain images that clearly delimited the walls of the aorta and its details. It was therefore possible to perform endovascular simulation, positioning the demonstration sample of the occluder device and of the bioprosthesis correctly, followed by their deployment.

During the procedure, the viewing angles and images were used to determine the best angiographic angles for optimal viewing of the relevant structures. Since each angiogram was optimally oriented, repeated angiograms were avoided, saving time, X-rays for the operators, and intravenous contrast for the patient.

## Therapeutic Intervention

**Figure 3:**
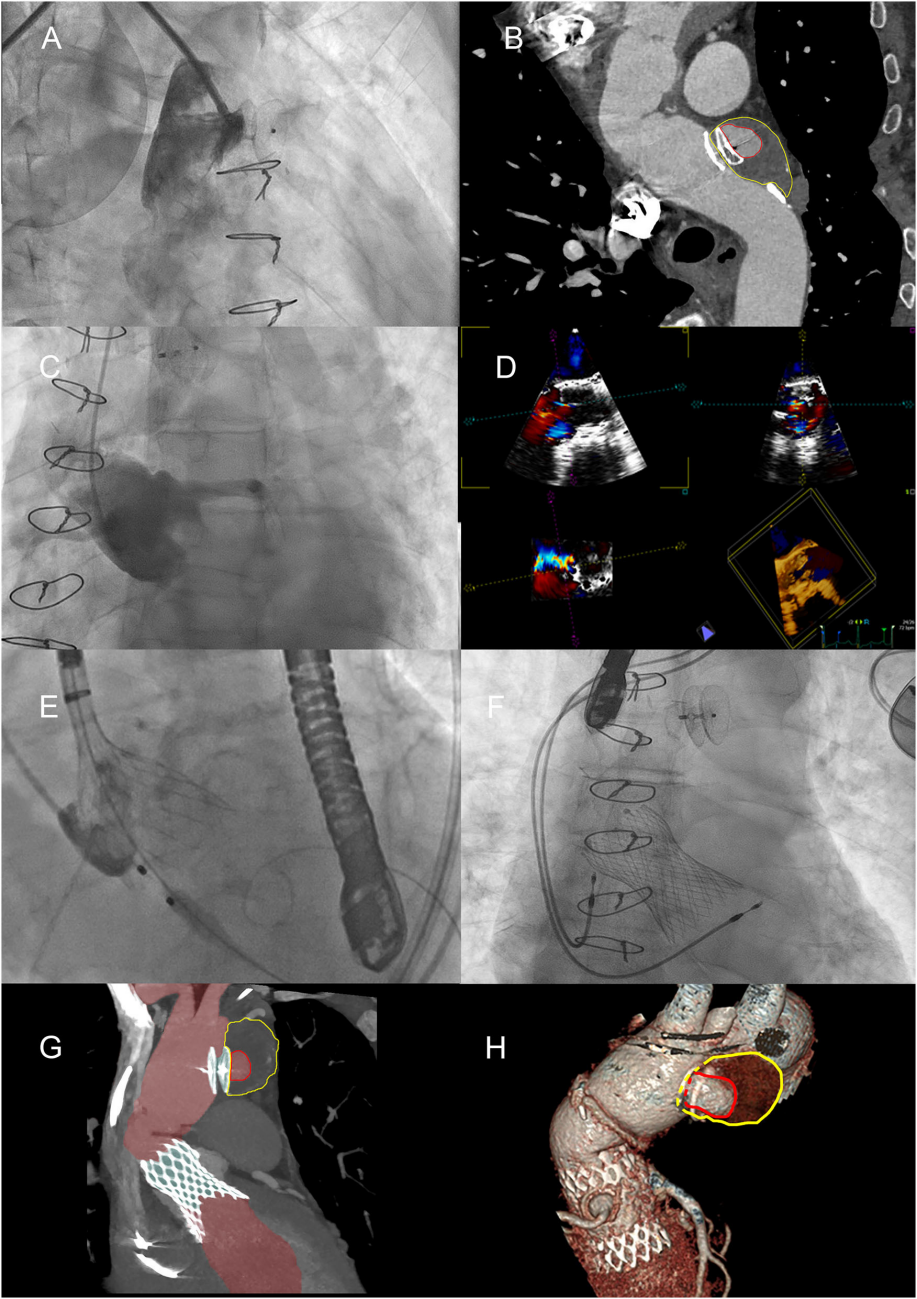
Positioning of the Amplatzer device and the Medtronic valve. **(A)** Positioning of the Amplatzer device, with angiography demonstrating the apparent total obliteration of the AAP. **(B)** MPR curved reconstruction of angio-CT, 1 month after placing the Amplatzer device. The red line highlights the perfused part of the AAP; the yellow line highlights the thrombosed part. **(C)** Severe failure of the native aortic valve, confirmed by angiography. **(D)** Study of the native aortic valve and confirmation of its severe insufficiency with transesophageal ultrasound. **(E)** Medtronic valve positioning by accurately locating the position of the annulus. **(F)** One-year later research for endoleak by transesophageal ultrasound. **(G)** Maximum intensity projection (MIP) reconstruction of angio-CT after placement of TAVI. The perfused part of the aorta and AAP is highlighted in red. **(H)** VR reconstructions of the partially thrombosed AAP.

After balancing the risks and benefits of all approaches (naturally including aortic angiography—Figures 2G,H), we decided to perform, with the patient under local anesthesia and via right brachial artery access (just above the cubital fossa of the elbow) with an eight-French sheath, percutaneous occlusion of the AAP with Amplatzer Septal Occluder 25-mm device (St. Jude Medical, Minneapolis, MN). The device was successfully implanted with the distal disk into the pseudoaneurysm and the proximal disk in the ascending aorta (Figure 3A). The whole process lasted a few minutes and caused minimal vessel injury. After a month of follow-up, CT scan confirmed stabilization of the device but with a small residual flow anterosuperior to the device (4 ml), with incomplete occlusion of the AAP (diameter 5.7 × 2.6 × 4.0 cm, with depressurized volume 21 ml; perfused diameter 2.8 × 1.3 × 2.5 cm, volume 4 ml) (Figure 3B).

The patient (with lesion-free coronary arteries) underwent TAVI procedure with Evolut R 34-mm valve (Medtronic, Minneapolis, MN) after testing the valve in a simulated reconstructed aortic root to ensure that its functional part was not impinged, as both the distal and proximal portions of the Evolut R would be restrained in this 7.5-cm graft. Moreover, both staged procedures were guided by intraoperative transesophageal echocardiography (TEE) (Figures 3C–E). Two days after the procedure, for the onset and persistence of third-degree atrioventricular block, the patient was required to implant a permanent pacemaker (DDDR pacing mode) (Figure 3F). The patient was discharged 3 days later.

**Figure 4:**
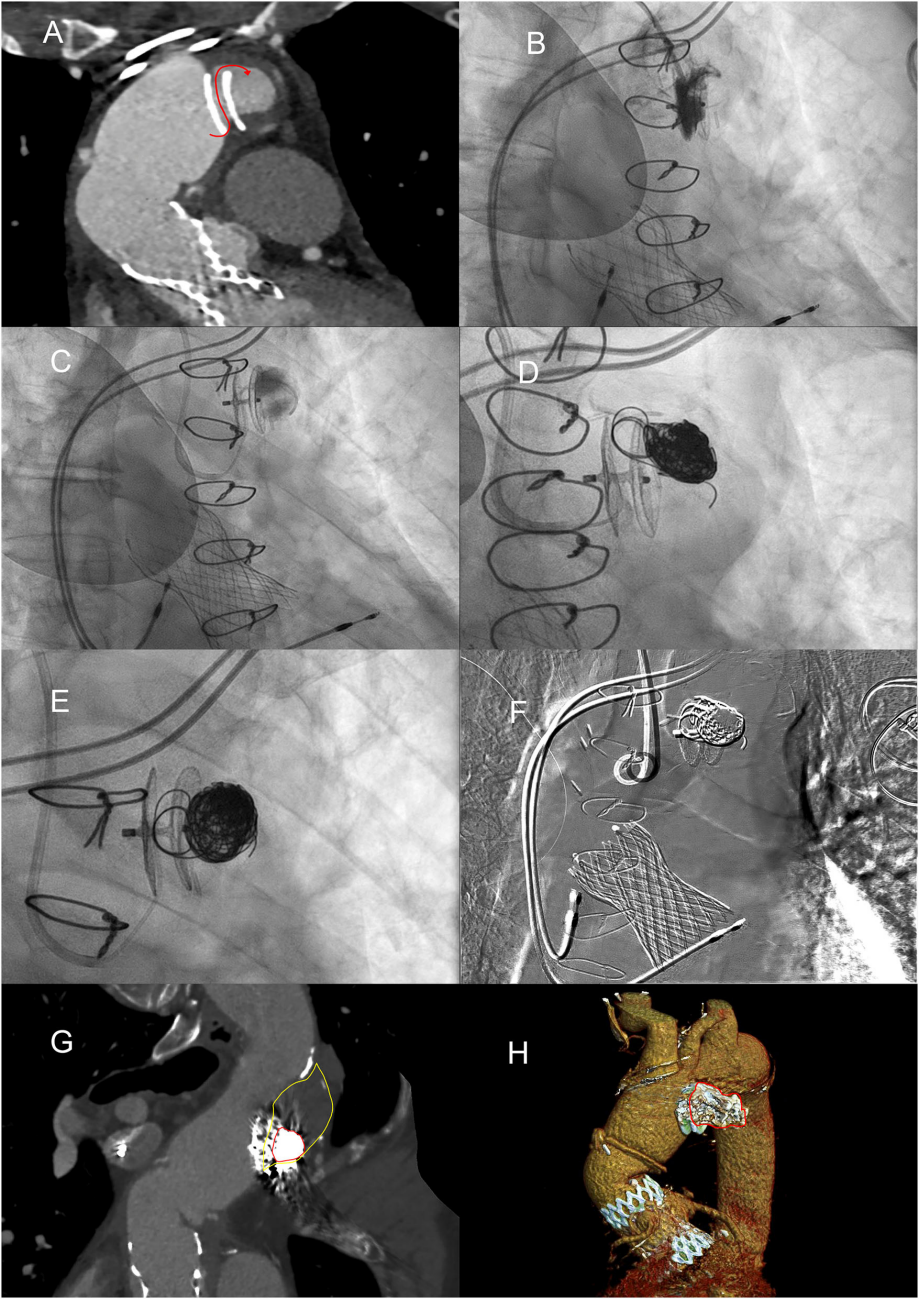
Embolization of the residual pseudoaneurysm, in volumetric expansion. **(A)** MPR reconstruction demonstrates continuity between the aortic lumen and the pseudoaneurysm lumen with flow direction highlighted by the red curved arrow. **(B)** Found the direction of blood flow with selective angiography, after performing CT and during transesophageal ultrasound. **(C)** Angiography demonstrating the opacification with microcatheter of the residual pseudoaneurysm sac. **(D)** Controlled release of coils by means of a microcatheter. **(E)** AAP completely filled with platinum embolization coils, enlaced with poly-lactide-co-glycolide acid (PGLA) fibers. **(F)** Digital subtraction angiography showing all “metallic” devices. **(G)** MPR curved reconstruction from angio-CT after placement of coils in the lumen of the AAP. **(H)** Angio-CT VR reconstructions of the thrombosed pseudoaneurysm with coils.

After 1 year, a CT scan showed the persistence of a residual peri-prosthetic flow, equal to a volume of about 3 ml, along the anterior profile of the Amplatzer device, but remodeling of the AAP was recognized with a total volume increasing by about 25% (from 21 to 28 ml), with a diameter of 6 × 2.8 × 4.1 cm and a perfused diameter of 2.2 × 1.4 × 1.8 cm (**Figures 3G,H, 4A**).

An aortography via the right radial artery confirmed the residual sac in AAP, anterosuperior to the Amplatzer (Figure 4B). Using a MP 6-Fr catheter, a microcatheter was placed in the sac, which was confirmed and opacified with radio-opaque contrast agent (Figure 4C). Then, the residual pseudoaneurysm was embolized using six controlled release platinum coils (Concerto Ev3, MRN compatible) (Figures 4D–F). The effective definitive closure of the residual pseudoaneurysmatic sac was confirmed with angiography and, after 1 month, with a CT scan (Figures 4G,H). The calculated volume of the positioned spirals was equal to 4 ml, equal to the residual volume of the perfused portion of the AAP.

For the three interventions (closure of APP, release coils, and TAVI), the mean operation time was 3.04 h. The total contrast agent volume was 405 ml, and the total radiation dose was 1,142 mGy. There was no significant blood loss or transfusion.

## Discussion

Clinical reports cannot be interpreted as a successful AVS when their evaluation is based exclusively on a freedom from reoperation. The results may not represent what happens after surgery because of the different outcomes of AVS in patients with various problems. Besides, there are only a few reports on outcomes beyond 10 years (6–9).

In this clinical case, we have two of the most fearful complications of AVS operation. The first is AI, which can lead to severe episodes of heart failure and delay treatment in a patient with COPD + OSAS. The second is AAP, often resulting from surgeries that involve cannulation of ascending aorta. Clinical presentation can be highly variable, but the former carries a high risk of pulmonary edema, whereas the latter a rupture of the AAP.

Endovascular/percutaneous repairs with occluder devices (10–12) and TAVI are used mainly in patients for whom major surgery is contraindicated. However, one of the disadvantages of percutaneous procedures is the mismatch between the deployed device and the waist of pseudoaneurysm or between the size of the biological valve and native aortic root. Dislocation of the occluder into the lumen and severe paravalvular leak can be avoided with the use of reproducing 3D models depicting thoracic aorta and three-dimensional TEE. Both multimodality imaging enabled us to confidently visualize and position the occluder device and valve and to assess them before a prerelease. These multimodality imagings, with the help of demonstration samples into the 3D printed model, could ultimately enhance preoperational planning and alleviate some of the risk of intra- and post-operative complications of associated therapies. In our case, we were able to demonstrate the usefulness of the 3D model for groundwork or fluoroscopic simulations and showed that the 3D model was very helpful for preoperative planning and orientation (role of valve tilt/inclination during placement and implantation depth with respect to the kneeling of the Dacron prosthesis), as well as to simulate procedures due to the exact and life-like illustration of the cardiovascular anatomy.

The 3D printed model proved useful in the discussion with the surgeons, it is complementary to the CT volume rendering reconstructions, and it allows us to define the sequencing of the implants. Last but not least, it serves as training and as a means of informed patient consent.

In the literature, some clinical cases have been well-described and brilliantly resolved with pre-fenestrated stent grafts to treat aortic arch disease (13). This treatment is undoubtedly a valid option, but always if there is the presence of an experienced team and after evaluating whether the anatomy is favorable. However, the authors (13) mainly describe cases of patients with single pathologies of the aortic arch (or dissections or aneurysms or pseudoaneurysms). Moreover, it is necessary to consider extending the collegial discussion also to vascular surgeons, consider the high costs and the risks related to vascular endoprostheses, and above all evaluate the time (procedural and X-ray exposure). In addition, blood loss and the volume of contrast agent are also factors to consider.

Our procedure demonstrated that with the creation of a 3D printed model and the choice of the best prostheses/devices, we were able to save time, contrast agent, and dose of X-ray.

## Patient Perspective

We successfully performed an AAP occlusion and bioprosthesis implantation in a patient with aortic valve-sparing operation. Complications can be avoided with multimodality imaging, also with 3D reconstruction, first by using a 3D printed model and then through a 3D TEE during the percutaneous procedures. The device and the bioprosthesis are a lifelong implant and do not necessarily need post-operative anticoagulation, but only single antiplatelet therapy. The Amplatzer device is a good and safe option for endovascular treatment given the right anatomy, and the combination with coil embolization made it possible to have immediate success and a high rate of complete occlusion of AAP, even in cases of partial failure with the occluder device (14).

After only a short follow-up, CT scan confirmed complete occlusion of AAP, while transthoracic echocardiography only detected a minimal paravalvular leak.

However, long-term follow-up is needed to confirm durability or survival. We have included the patient in a follow-up program, so as to evaluate him periodically in an outpatient clinic dedicated to structural diseases.

## Data Availability Statement

The raw data supporting the conclusions of this article will be made available by the authors, without undue reservation.

## Ethics Statement

Written informed consent was obtained from the individual(s) for the publication of any potentially identifiable images or data included in this article.

## Author Contributions

LV, LS, AD, VS, MS, EC, PM, and FC contributed to the writing and proofreading of the manuscript. All authors contributed to the article and approved the submitted version.

## Conflict of Interest

The authors declare that the research was conducted in the absence of any commercial or financial relationships that could be construed as a potential conflict of interest.

## Publisher's Note

All claims expressed in this article are solely those of the authors and do not necessarily represent those of their affiliated organizations, or those of the publisher, the editors and the reviewers. Any product that may be evaluated in this article, or claim that may be made by its manufacturer, is not guaranteed or endorsed by the publisher.
